# Cytomegalovirus Retinitis and Retinal Detachment following Chimeric Antigen Receptor T Cell Therapy for Relapsed/Refractory Multiple Myeloma

**DOI:** 10.3390/curroncol29020044

**Published:** 2022-01-24

**Authors:** Cheng Zu, Yufeng Xu, Yiyun Wang, Mingming Zhang, Houli Zhao, Xiaoyun Fang, He Huang, Yongxian Hu

**Affiliations:** 1 Bone Marrow Transplantation Center, The First Affiliated Hospital, Zhejiang University School of Medicine, Hangzhou 310003, China; wallczu@zju.edu.cn (C.Z.); 11918268@zju.edu.cn (Y.W.); mingmingzhang@zju.edu.cn (M.Z.); 11918563@zju.edu.cn (H.Z.); 2 Institute of Hematology, Zhejiang University, Hangzhou 310058, China; 3 Zhejiang Province Engineering Laboratory for Stem Cell and Immunity Therapy, Zhejiang University, Hangzhou 310058, China; 4 Zhejiang Laboratory for Systems & Precision Medicine, Zhejiang University Medical Center, Hangzhou 311100, China; 5 Eye Center, The Second Affiliated Hospital, Zhejiang University School of Medicine, Hangzhou 310009, China; xuyufeng0216@zju.edu.cn (Y.X.); fangxiaoyun@zju.edu.cn (X.F.)

**Keywords:** cytomegalovirus infection, retinitis, retinal detachment, chimeric antigen receptor T cells, multiple myeloma

## Abstract

Cytomegalovirus (CMV) retinitis is a rare end-organ disease of CMV infection and is a marker of severe immunosuppression, especially in human immunodeficiency virus (HIV)-positive patients. In multiple myeloma (MM) patients, CMV retinitis has been reported in the post-transplant setting, with an incidence lower than 0.2%, and in patients receiving lenalidomide. Here, we describe the first case of CMV retinitis in myeloma patients following B-cell maturation antigen (BCMA)-targeted chimeric antigen receptor T (BCMA CAR-T) cell therapy. In addition to CMV, the patient developed multiple infections including a mouth ulcer, pneumonia, and fungal enteritis. While the complete remission (CR) status of MM was maintained, he regained a visual acuity of 20/1000 after appropriate ophthalmologic treatment. This single case illustrates the potential of BCMA CAR-T therapy to induce profound humoral immunosuppression, and demonstrates an imperative need for an established standard of monitoring and prophylaxis of post-CAR-T infections.

## 1. Introduction

Cytomegalovirus (CMV) is an opportunistic pathogen that may cause a wide spectrum of clinical manifestations in immunocompromised patients, ranging from the more common gastrointestinal disease to rare retinitis and encephalitis [1]. The term immunocompromised patients generally refers to human immunodeficiency virus (HIV)-positive patients with extremely low CD4^+^ T cell counts [2], while in the hematology patient population, CMV retinitis is usually related to allogeneic hematopoietic stem cell transplantation (allo-HSCT) [3], although this is uncommon, and post-autologous hematopoietic stem cell transplantation (auto-HSCT) CMV retinitis has been reported in recent years [4].

Chimeric antigen receptor T (CAR-T) cell therapy is a novel adoptive immunotherapy which involves genetic engineering of T lymphocytes. These genetically modified T cells carry a chimeric antigen receptor (CAR) that targets tumor antigens. For multiple myeloma patients, B-cell maturation antigen (BCMA) is an ideal target of CAR-T therapy, except for the on-target/off-tumor toxicity against normal circulating plasma cells [5]. The consequential aplasia of plasma cells and hypogammaglobinemia [6] may further exacerbate the inherent immune dysfunction in the patient population who receive CAR-T therapy [7].

Here, we describe a patient who developed CMV retinitis and consequential retinal detachment following BCMA-targeted CAR-T cell therapy against multiple myeloma, which, to our best knowledge, is the first case in this patient population [7].

## 2. Case Description

A 58-year-old male was diagnosed with multiple myeloma (IgA-κ, Durie-Salmon staging system (DS) stage IIA, revised international staging system (R-ISS) stage I) in March 2019 based on extensive bony lytic lesions, monoclonal IgA, and free light chain (FLC)-κ detected by immunofixation electrophoresis in both serum and urine, and proplasmacytes in bone marrow (BM). Fluorescence in situ hybridization (FISH) revealed no cytogenetic abnormality. Following four cycles of a bortezomib–dexamethasone–lenalidomide (VRD) chemotherapy regimen, he was assessed as very good partial response (VGPR) in July 2019. He declined our suggestion of auto-HSCT to further consolidate the response. Two months later, bone marrow aspiration showed the reappearance of proplasmacytes (5%), indicating the relapse of MM, and the serum immunofixation electrophoresis confirmed it with monoclonal IgA and FLC-κ. Despite several more courses of chemotherapy (including two cycles of VRD, two cycles of cyclophosphamide–etoposide–cisplatin–dexamethasone (DECP), and three cycles of daratumumab–melphalan–dexamethasone–thalidomide (DARA+MPT)), the patient presented unbearable bone pain throughout the body, which was further confirmed to be the result of osteolytic lesions by MM, with extremely elevated levels of serum IgA (1314 mg/dL) and FLC-κ (78.7 mg/dL), as well as sustained proplasmacytes (6%) at the time of admission. In order to control the progression of MM, the patient was enrolled in a clinical trial (ChiCTR.org.cn (accessed on 1 September 2021), ChiCTR1800017404), which involved a course of B-cell maturation antigen (BCMA) CAR-T cell therapy, after the approval by the ethics committee of the First Affiliated Hospital of Zhejiang University School of Medicine.

Following the manufacture of CAR-T cells and a 3-day preconditioning fludarabine–cyclophosphamide (FC) chemotherapy, he received BCMA CAR-T cells at a dose of 1.66 × 10^8^ cells (2.72 × 10^6^ cells/kg) in June 2020.

Unfortunately, in addition to the common adverse events related to CAR-T therapy, such as cytokine release syndrome (CRS), pancytopenia (especially neutropenia), and neurotoxicity, he developed a mouth ulcer, pneumonia, and fungal enteritis before discharge (more detailed information about adverse events is listed in Table 1). He did not receive steroids even though the CRS was relatively severe (Grade 3), tocilizumab (anti-IL-6R monoclonal antibody) was prescribed instead. For the treatment of present infections and the prevention of potential infections, we used multiple antibiotics including piperacillin/tazobactam, cefoperazone/sulbactam, vancomycin, biapenem, fluconazole, caspofungin, and ornidazole, while antiviral prophylaxis was neglected. Intravenous immunoglobulin (IVIg) support was considered unnecessary because of a stable level of IgG (range: 210–249 mg/dL) in the first month after CAR-T. Furthermore, tests for CMV, either before or after CAR-T therapy, are not routine in our center.

**Figure 1:**
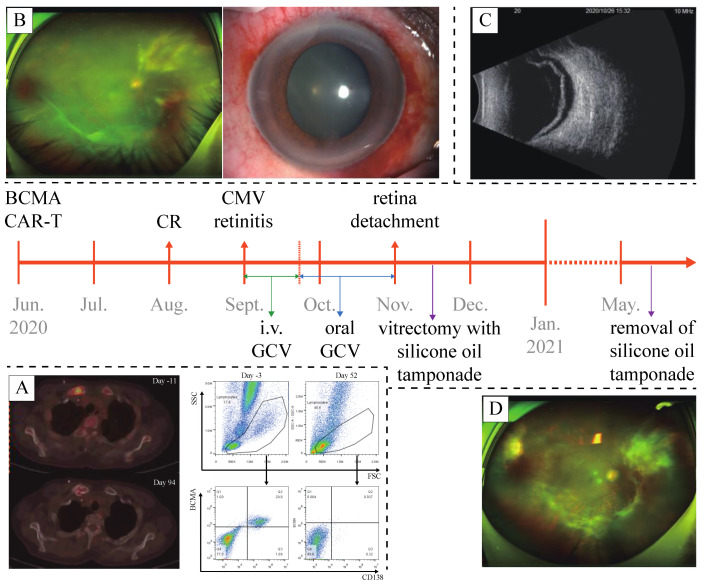
A timeline of the case. (**A**) Post-CAR-T flow cytometry of bone marrow sample and PET-CT confirmed the complete remission of multiple myeloma. (**B**) Anterior segment examination and fundoscopy at the diagnosis of CMV retinitis. (**C**) Ultrasonography of the right eye confirmed retinal detachment. (**D**) Fundoscopy after the removal of silicone oil tamponade, showing recovery from CMV retinitis and retinal detachment.

With the disappearance of osteolytic soft tissues, the clearance of abnormal plasma cells in the BM (Figure 1A), and the decrease in serum FLC-κ (0.2 mg/dL) and IgA (16 mg/dL) levels, he was assessed as being in complete remission (CR) with minimal residual disease (MRD)-negative 52 days after the infusion, and has maintained CR status until the writing of this article (August 2021). However, in September 2020, he reported decreasing visual acuity in his right eye and presented for further investigation.

On examination by an ophthalmologist, the visual acuity in his right eye was 20/800, mutton-fat keratic precipitates (KP), aqueous flare and cell, and post-synechia were revealed in anterior segment examination. The assessment was unable to acquire a clear view of the fundus resulting from his cloudy vitreous body, but an abnormal patch of retinitis was vaguely identified (Figure 1B). Examination of the fellow eye was unremarkable. A sample of vitreous humor from the right eye was then acquired for next-generation sequencing (NGS), which revealed the presence of CMV DNA. Given a normal level of lymphocytes (3.2 × 10^9^/L), the subpopulations of T lymphocytes were not further investigated. As soon as the diagnosis of CMV retinitis was established, a course of intravenous ganciclovir (0.3 g b.i.d.) for 3 weeks was administered. The continuous follow-up showed a considerable increase in the visual acuity in his right eye (20/25), anterior segment examination revealed no remarkable finding, and fundoscopic photography also showed resolution of the retinitis lesion. Thus, instead of intravenous administration, oral ganciclovir (0.5 g t.i.d.) was prescribed for consolidation.

Unfortunately, the patient’s visual acuity in his right eye decreased to light perception in October 2020. Anterior segment examination found nothing remarkable, fundoscopic photography was unable to obtain a clear view. Optical coherence tomography (OCT) and ultrasonography (US) revealed retinal detachment in the right eye (Figure 1C). Vitrectomy with silicone oil tamponade was then performed. Post-operational OCT and US confirmed the repositioning of the retina. Seven months later, in May 2021, silicone oil removal was performed along with phacoemulsification and intraocular lens implantation for the treatment of a complicated cataract. Eventually, the visual acuity in the patient’s right eye was restored to 20/1000. Fundoscopic photography, OCT, and anterior segment examination all confirmed the recovery from CMV retinitis and retinal detachment (Figure 1D).

## 3. Discussion

In this report, we described a patient who developed CMV retinitis 3 months following BCMA CAR-T therapy against relapsed MM with no evidence of other end-organ involvement. Since whether he had a latent CMV infection prior to CAR-T therapy was unclear, we would not like to draw an arbitrary conclusion, but CMV retinitis is generally believed to result from the systemic reactivation of a latent infection, which explains the relatively high incidence in immunosuppressed patients [8]. In myeloma patients, CMV reactivation is almost exclusively associated with auto-/allo-HSCT, usually with viremia only [9,10]. Among all CMV end-organ diseases, CMV retinitis is the rarest. Crippa et al. identified only 10 cases of CMV retinitis among 5721 patients who received allo-/auto-HSCT in a 14-year period [10]. They described CMV retinitis as a late-onset complication (median 251 days post-HSCT) [10].

The treatment of MM has undergone a paradigm shift with increasing use of therapies targeting the immune system, including immunomodulatory drugs (IMiDs) and monoclonal antibodies (mAbs). Cases of CMV retinitis and other end-organ diseases related to the use of IMiDs (e.g., lenalidomide) and mAbs (e.g., daratumumab) have been reported [11,12], and it seems that CAR-T cell therapy is no exception.

A number of factors are assumed to play a role in the increased risk of infection in patients undergoing CAR-T cell therapy, including the inherent immune dysfunction of hematological malignancies, multiple prior lines of therapy, lymphodepletion chemotherapy, immune-suppressing agents against complications (e.g., CRS, immune effector cell-associated neurotoxicity syndrome [ICANS], etc.), prolonged cytopenia, on-target/off-tumor toxicity against normal constituents of immune system, etc. Compared to bacterial infections, viral infection is of less incidence, but of no less importance. Reactivation of latent virus infections after CAR-T therapy, including hepatitis B virus (HBV), Epstein–Barr virus (EBV), CMV, etc., has been identified in various studies [13,14,15,16,17]. While most of the reactivations caused no end-organ disease, severe infection such as progressive multifocal encephalopathy has been reported [18].

To some extent, most attention of the researchers who are trying to push CAR-T therapy forward has been drawn to the life-threatening complications such as CRS and ICANS. In contrast, the understanding of post-CAR-T infections is lacking, the current prophylaxis and management are largely extrapolated from the experiences of auto- and allo-HSCT [7]. In recent years, we have seen a few studies reflecting on the infectious complications following CAR-T therapy, mostly retrospective [13,14,15,16,17]. The rates of early infection (<30 days) range from 17% to 42%, with the predominance of bacterial infections; while the rates of late infection (>30 days) range from 14% to 31%, with the predominance of viral infections [13,14,15]. Though of slightly lower incidence, viral infection is of no less importance. Severe viral complication such as JC virus-related progressive multifocal leukoencephalopathy (PML) has been reported [18], as well as the reactivation of other latent viral infections (e.g., CMV, EBV, and etc.) with a few end-organ diseases [13,14,15,16,17]. Given the observation of viral reactivation [13,14,15,16,17], and the potential need for CAR-T therapy in the massive patient population with chronic viral infections (e.g., hepatitis B virus (HBV), hepatitis C virus (HCV), human immunodeficiency virus (HIV), etc.) [19,20,21,22,23], viral infection is one of the top problems to be addressed in CAR-T cell therapy.

In regard of CMV, it was detected in the plasma of 7 patients out of a total of 435 patients in the CAR-T studies mentioned above, with no end-organ disease reported [13,14,15,16,17]. Since the test for CMV was only clinically driven in these studies, the lack of routine screening for CMV may contribute to the low incidence. Although the incidence is low, CMV can be seriously damaging, even fatal in both immunocompetent and immunosuppressed patients [24]. After decades of exploration, CMV prevention with antiviral agents and immune monitoring for CMV have become routine in both HSCT and solid organ transplantation [1,25]. Given the heavy pretreatment of CAR-T recipients (notably, one-third of whom had had prior allo-HSCT), it could be expected that the rate of CMV reactivation would be relatively considerable [7]. In that case, further interventions similar to those that are applied now in post-transplant patients should be taken into consideration. Taking into account the late-onset and low-incidence nature of CMV infection/reactivation after CAR-T, active and long-term antiviral administration does not seem an economic strategy. Instead, we believe that a routine screening for CMV for CAR-T cell recipients, both pre- and post-infusion, is more cost-effective and necessary.

Apart from CMV, other opportunistic infections, such as fungal enteritis in our patient, VZV reactivation with retinitis, EBV viremia, etc., in the post-CAR-T setting are emerging [15,26]. It is predictable that with the expanding application of CAR-T cells, as well as the evolving management of CRS and ICANS, especially immunosuppressive drugs, infectious complications are coming to the center of the stage. Here, we are calling upon all our peers to work together to further understand the patterns, risk factors, clinical features, diagnosis, prevention strategies, management, etc., of post-CAR-T infections. The numerous patients treated by CAR-T therapy are a rich reservoir for retrospective study, but it is difficult to interpret the role of heterogenous anti-infectious strategies they received because of the epidemiological and institutional differences. Therefore, active studies on the role of screening and prophylaxis specific to CAR-T cell therapy are needed to enlighten clinical practice.

## Figures and Tables

**Table 1:** Detailed adverse events of the patient.

Adverse Events	Grade
**CRS** (Tocilizumab used)	3
**Neurological**	
Peripheral motor neuropathy (Convulsion in both lower limbs)	2
Peripheral sensory neuropathy (Pain in both lower limbs)	2
**Hematological**	
Anemia	3
Thrombocytopenia	2
Febrile neutropenia	3
**Infectious**	
Enterocolitis infectious (Fungal enteritis)	3
Cytomegalovirus infection reactivation (CMV retinitis)	3
Mucositis oral (Ulcerations)	3
Lung infection	3
**Others**	
Alanine aminotransferase increased	1

Abbreviations: CRS, cytokine release syndrome; CMV, cytomegalovirus. The grading of adverse events was based on *the Common Terminology Criteria for Adverse Events 5.0 (CTCAE 5.0)*.

## Data Availability

The data presented in this study are available on request from the corresponding authors. The data is not publicly available due to the privacy of the patient.
